# Inflammatory cytokines secreted from senescent periodontal ligament cells influence the osteocyte network in alveolar bone

**DOI:** 10.1093/jbmrpl/ziag014

**Published:** 2026-01-29

**Authors:** Akimi Yamashita, Hirofumi Tenshin, SooHa Matsuki, Go Nishino, Shinetsetseg Ser-Od, Filippo Moenne Saito, Masahiro Hiasa, Eiji Tanaka

**Affiliations:** Department of Orthodontics and Dentofacial Orthopedics, Tokushima University Graduate School of Oral Sciences, Tokushima, 770-8504, Japan; Department of Orthodontics and Dentofacial Orthopedics, Tokushima University Graduate School of Biomedical Sciences, Tokushima, 770-8504, Japan; Department of Orthodontics and Dentofacial Orthopedics, Tokushima University Graduate School of Biomedical Sciences, Tokushima, 770-8504, Japan; Department of Orthodontics and Dentofacial Orthopedics, Tokushima University Graduate School of Oral Sciences, Tokushima, 770-8504, Japan; Department of Orthodontics and Dentofacial Orthopedics, Tokushima University Graduate School of Oral Sciences, Tokushima, 770-8504, Japan; Department of Orthodontics and Dentofacial Orthopedics, Tokushima University Graduate School of Oral Sciences, Tokushima, 770-8504, Japan; Department of Orthodontics and Dentofacial Orthopedics, Tokushima University Graduate School of Biomedical Sciences, Tokushima, 770-8504, Japan; Department of Orthodontics and Dentofacial Orthopedics, Tokushima University Graduate School of Biomedical Sciences, Tokushima, 770-8504, Japan

**Keywords:** lacunar-canalicular network, osteocyte, senescence-associated secretory phenotype, senotherapeutics, *podoplanin*/E11

## Abstract

The rate of orthodontic tooth movement slows with age, likely due to changes in alveolar bone remodeling. However, the underlying mechanisms remain poorly understood. Osteocytes (OCys), which form the lacunar-canalicular network (LCN), play a key role in bone metabolism; however, the effects of aging on the alveolar LCN are still unclear. This study aimed to clarify age-related alterations in the LCN of alveolar bone and their underlying mechanisms. The LCN was visualized using Ploton silver staining, revealing a significant reduction in 16-mo-old C57BL/6J mice compared to 2-mo-old mice. The number of OCys, as well as the number and length of canaliculi per OCy, decreased significantly with age. These reductions were consistent in both sexes. Periodontal ligament cells (PDLCs) from aged mice showed increased expression of the senescence marker p16^INK4A^ and upregulation of senescence-associated secretory phenotype (SASP) factor mRNAs. To evaluate the effect of SASP factors on LCN formation, a 3D-block culture system was utilized. Conditioned medium (CM) from senescent human PDLCs (over 20 passages) suppressed dendrite formation in the 3D culture as well as the expression of *E11/podoplanin*, while CM from young PDLCs had no significant effect. To test whether SASP inhibition could preserve LCN, the senolytic agents Dasatinib and Quercetin (D + Q) were administered to 12-mo-old mice for 4 mo. D + Q treatment significantly increased both canalicular number and length per OCy in alveolar bone compared to vehicle controls. In addition, D + Q treatment increased E11 expression and suppressed sclerostin expression on OCys in aged alveolar bones. These findings suggest that SASP factors from senescent PDLCs contribute to the age-related LCN deterioration in alveolar bone, potentially impairing OCy function and bone remodeling. Targeting PDL cellular senescence may therefore offer a novel strategy for preserving alveolar bone integrity with aging.

## Graphical Abstract

**Graphical Abstract:**
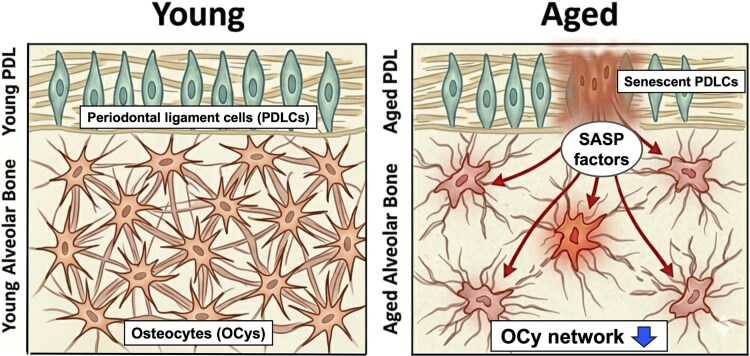

## Introduction

Maintaining better oral function is crucial for extending healthy life expectancy.^1,2^ The alveolar bone has the unique ability to sense mechanical loads through the teeth and periodontal ligament (PDL).^3^ Mechanical loading, including masticatory and orthodontic forces, stimulates alveolar bone remodeling and plays a critical role in maintaining bone homeostasis as well as facilitating orthodontic tooth movement.^4,5^ Aging is associated with various changes in the alveolar bone, such as increased susceptibility to infection,^6^ decreased bone volume,^7^ and reduced responsiveness to orthodontic forces.^8^ However, the mechanisms underlying age-related alterations in alveolar bone remodeling remain largely unclear.

Osteocytes (OCys) form a complex lacunar-canalicular network (LCN) through dendritic processes and play a vital role in sensing mechanical stress and regulating both local and systemic bone metabolism.^9,10^ In long bones, age-associated reductions in the LCN have been demonstrated in both animal and human studies.^11–13^ These changes include a decrease in the number and connectivity of canaliculi, leading to impaired fluid dynamics, decreased shear stress, and limited mechanosensitive.^14,15^ Such degeneration has been shown to negatively affect OCy-mediated signaling and bone remodeling capacity.^16^ Nevertheless, the precise mechanisms of LCN reduction with aging remain elusive.

While many studies have investigated LCN aging in long bones, few have examined alveolar bone aging. The alveolar bone differs from the long bone in terms of its embryonic origin, microstructure, and response to mechanical stimuli.^17^ Thus, analyzing age-related changes in the alveolar bone LCN and their mechanisms is indispensable to understanding impaired alveolar bone remodeling with aging. Recently, it was reported that senescent PDL cells (PDLCs) produce more inflammatory cytokines via the senescence-associated secretory phenotype (SASP) in aged PDL.^18^ Senescence-associated secretory phenotype causes chronic inflammation in the microenvironment,^19^ and prolonged exposure to SASP alters the characteristics of surrounding cells.^20,21^ Since the inflammation interrupts OCy maturation and dendrite processes,^22^ these SASP factors may contribute to the alterations in the LCN in the aged alveolar bone.

Therefore, this study aimed to clarify age-related alterations in the LCN of the alveolar bone and to investigate the mechanisms underlying the age-related decline in alveolar bone remodeling capacity. We analyzed structural changes in the LCN of the alveolar bone in both young and old mice of both sexes and found a significant reduction in LCN in old mice. In aged alveolar bone, PDLCs exhibited upregulated expression of the senescence marker p16^INK4A^, along with increased mRNA levels of SASP factors. Additionally, MLO-Y4 OCy-like cells exhibited reduced dendritic formation in a three-dimensional (3D) culture system supplemented with conditioned medium (CM) obtained from aged PDLCs. Notably, the administration of a senolytic cocktail comprising Dasatinib and Quercetin (D + Q) mitigated the age-related reductions in LCN and E11 expression. These results indicate that SASP factors secreted by senescent PDLCs may contribute to the deterioration of the LCN observed in aged alveolar bone.

## Materials and methods

### Antibodies and reagents

The following reagents were purchased from the indicated manufacturers: anti-p16^INK4A^ antibody (ab211542) from abcam; anti-E11/podoplanin antibody (AF3244-SP), anti-SOST/sclerostin antibody (AF1589), rabbit control IgG (AB-105-C), goat control IgG (AB-108-C) from R&D Systems; anti-Cathepsin K (CTSK) antibody (11239-1-AP) from Proteintech; Dasanitib (SML2589) and Quercetin (Q4951) from Sigma-Aldrich.

### Animal studies

Animal experiments were performed under the regulation and with the permission of the Animal Care and Use Committee of Tokushima University, Tokushima, Japan (T2024-16). The animal studies comply with the ARRIVE 2.0 protocol. Male and female C57BL/6J mice were purchased from SLC at 2, 12, or 16 mo of age. Mice were kept under regular light/dark cycles (12 h light:12 h dark) at a constant temperature (23 ± 2 °C). Mice were allowed to acclimate to the facility for 1 wk prior to any experimental procedures. For each experiment, a randomly selected cage containing up to 5 mice was assigned a random location within the animal facility to minimize potential confounding due to environmental factors. For D + Q treatment, 12-mo-old mice were randomly divided into either the vehicle control (VC) group or the D + Q-treated group. Randomization was performed using tables generated with the RAND function in Microsoft Excel. Mice were treated by oral gavage with D + Q or vehicle once every 2 wk for 4 mo. D + Q were diluted in 10% PEG400 and administered by oral gavage at doses of 5 and 50 mg/kg, respectively, in 100 μL, as previously described.^23^ Humane endpoints were defined based on animal welfare considerations, and animals exhibiting marked deterioration due to drug administration were to be euthanized; however, no animals met the criteria for euthanasia during the study. For the comparison between young and old mice, 2 independent experiments were performed (*n* = 3 in each group), and representative data are shown. For the comparison between the VC and D + Q-treated groups, 3 independent experiments were performed, each including 3-5 animals per group (*n* = 3-5 in each group), and representative results are presented. No animals were excluded. The blinded observer performed outcome measurements.

### Sample preparation of alveolar bones for histology

Mice were fixed by perfusion fixation using 4% paraformaldehyde (PFA). The maxillae were harvested and further fixed in 4% PFA for 24 h at 4 °C and decalcified using G-Chelate Mild (Genostaff) for 14 d at room temperature (RT). They were then embedded in paraffin. Longitudinal sections including maxillary first and second molars (5 μm) were cut and mounted on glass slides. Every staining was observed by a blinded observer.

### Ploton silver staining

Ploton silver staining was performed as previously described.^24^ Briefly, the sections were deparaffinized and hydrated before treatment with 50% silver nitrate solution for 55 min at RT. Sections were washed with 5% sodium thiosulfate for 10 min at RT and counter-stained with hematoxylin. After dehydration, sections were mounted and observed with a light microscope (BZ-X800, Keyence). Lacunar-canalicular network parameters were measured within a region of interest (ROI) defined as 200 μm of the PDL on the first-molar side in the alveolar bone between the distal root of the first molar and the mesial root of the second molar, from the alveolar crest to the level corresponding to the apex of the first molar. Within this ROI, the LCN area (mm^2^) was measured and expressed as a percentage of the bone area, as the LCN area per bone area (%). This ratio, as well as the canalicular number and length per OCy, was quantified using ImageJ software (NIH) according to previously described methods.^25,26^ For each mouse, the mean value was calculated from three randomly selected fields of view.

### Immunohistochemistry

The sections were deparaffinized and hydrated. After blocking endogenous peroxidase with 3% H_2_O_2_, antigen unmasking was performed for 20 min using the L.A.B. solution (24310, Polysciences, Inc.) at RT. Then, sections were treated with normal serum for 45 min at RT, followed by treatment with primary antibody against goat anti-E11 antibody (1/50), anti-sclerostin antibody (1/50), rabbit anti-CTSK antibody (1/200), or the same concentration of control IgG. The sections were incubated overnight and treated with Horseradish Peroxidase (HRP)-conjugated secondary antibody (ImmPRESS^®^ Reagent HRP, Vector Laboratories) before color development by 3,3'-Diaminobenzidine (DAB) solution (Takara Bio Inc.) and hematoxylin. The images were captured with the BZ-X800 microscope (Keyence), and the ratio of E11-, sclerostin-, or CTSK-positive OCys per total OCys was assessed in the alveolar bone within the same ROI used for the LCN measurement using ImageJ software (NIH).

### Immunofluorescence

The sections were deparaffinized and hydrated. Antigen retrieval was performed for 20 min using the L.A.B. solution (Polysciences, Inc.) at RT. Subsequently, the sections were treated with 1% BSA with 0.1% Triton X for 30 min at RT, followed by the addition of rabbit anti-p16^INK4A^ antibody (1/100) or control IgG. The sections were incubated overnight and treated with a secondary antibody, anti-rabbit IgG conjugated with Alexa Fluor 594 (1/100, 711-585-152, Jackson ImmunoResearch Laboratories, Inc.) for 1 h at RT. The sections were stained with Hoechst for 5 min (Hoechst 33342, Invitrogen). The images were captured with the BZ-X800 fluorescent microscope (Keyence), and the ratio of p16^INK4A^-expressing PDLCs per total PDLCs in the PDL around the distal root of the maxillary first molar was analyzed with ImageJ software (NIH).

### H&E staining and tartrate-resistant acid phosphatase (TRAP) staining

The sections were deparaffinized, hydrated, and stained with H&E following standard protocols. TRAP staining was performed on deparaffinized bone sections using the TRAP/Alkaline Phosphatase stain kit (Wako) according to the manufacturer’s instructions.

### Cell culture

The murine osteocyte-like cell line MLO-Y4 was kindly provided by Dr. Lynda F. Bonewald (Indiana University). Primary human PDLCs (hPDLCs) were purchased from LONZA (CC-7049). The cells were cultured in Eagle’s Minimal Essential Medium Alpha Modification (α-MEM) (Fujifilm Corporation) supplemented with 10% FBS and 50 mg/mL penicillin/streptomycin (Sigma-Aldrich).

### Isolation of CM from hPDLCs

Human PDLCs were serially passaged more than 20 times to induce cellular senescence according to the previous report.^18^ Human PDLCs passaged fewer than 10 times were defined as young hPDLCs. Young and aged hPDLCs were cultured in α-MEM media containing 10% FBS until they became confluent, and then the media were changed to α-MEM without FBS, and the cells were further cultured for 2 d. The culture supernatants collected from young and aged hPDLCs were concentrated by ultrafiltration using Amicon Ultra-15 Centrifugal Filter Units (MilliporeSigma) after removal of cellular components to generate CM. Young and aged CM were added at a 20% concentration in each experiment.

### 3D culture of MLO-Y4 cells

MLO-Y4 cells were seeded onto Bio-Block^27^ produced by RONAWK, Inc. at 1 × 10^6^ cells per block, referring to the manufacturer’s instructions. The cells were cultured for 5 d to allow sufficient attachment and growth within the block. Culture media were replaced with media containing CM from young and aged hPDLCs, and the cells were further cultured for 2 d. The blocks were fixed in 2% PFA containing 0.01% sucrose for 1 wk at 4 °C, according to the manufacturer’s instructions. The dendrites and cells in the fixed blocks were visualized with iFluor 488-conjugated Phalloidin (#20549, Cayman Chemical) and Hoechst (Invitrogen). The images were captured using z-stack mode with the BZ-X800 fluorescent microscope (Keyence), and dendrite length in the Bio-Block was quantified by tracing the longest process from each cell with ImageJ software (NIH).

### Real-time quantitative PCR

Total RNA was extracted using TRIzol reagent (Invitrogen). Two microgram of total RNA was reverse-transcribed with PrimeScript RT (Takara Bio Inc.) in a 10 μL reaction solution. To perform the real-time quantitative PCR (RT-qPCR), each cDNA sample was amplified using GeneAce SYBR qPCR mix (Nippon Gene Co., Ltd.) on the 7300 Real-time PCR System (Thermo Fisher Scientific). The reaction conditions consisted of 2 μL of cDNA and 0.4 μM primers in a total volume of 20 μL. *Hprt* was used as an endogenous control to normalize each sample. Melting curve analysis was used to confirm each primer pair produced a single product. Primer sequences are listed in Table S1.

### Statistical analysis

Statistical analysis was performed using the Student’s *t*-test for 2 groups. For multiple groups, statistical differences were assessed by one-way analysis of variance (ANOVA) with Tukey’s test. *p* < .05 was considered a significant difference. The sample size was estimated using preliminary results and power analysis. In this study, all statistical analyses were performed using Statcel 4 Software (OMS Publishing).

## Results

### LCN was decreased in the alveolar bone in old mice

**Figure 1:**
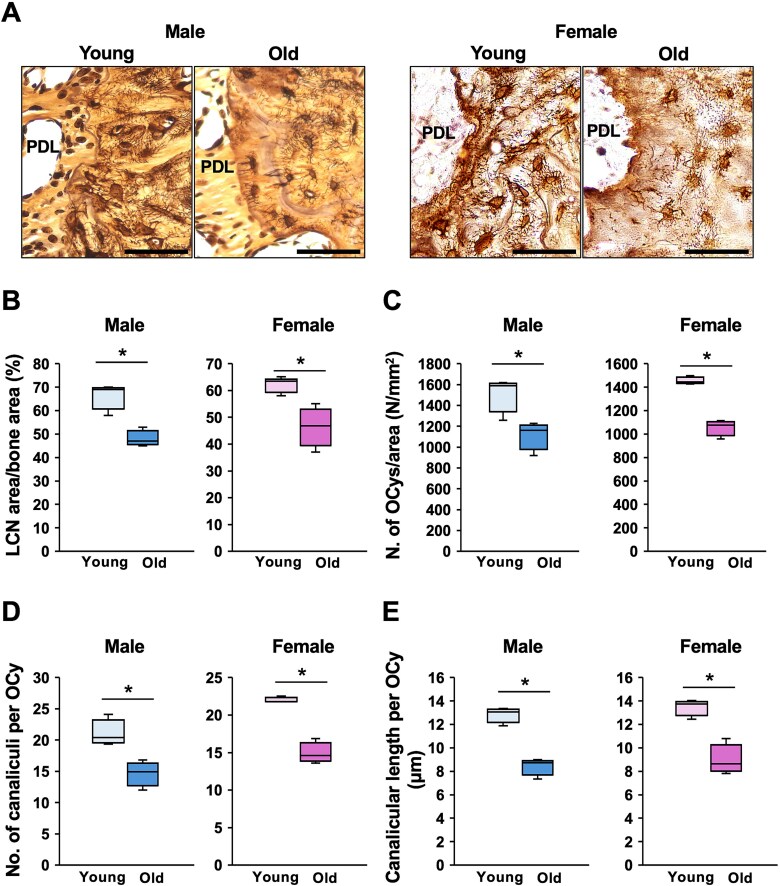
Lacunar-canalicular network (LCN) in the alveolar bone from young and old mice. Maxillary sections derived from 2-mo-old (young) and 16-mo-old (old) male and female mice were stained with Ploton silver staining. (A) Representative images of LCN in the alveolar bone from young and old mice are shown. Scale bars are 50 μm. PDL, periodontal ligament. (B) The LCN area per bone area was quantified in the alveolar bone between the distal root of the first molar and the mesial root of the second molar (*n* = 3 in each group). ^*^; *p* < .05 by *t*-test. (C) The number of OCys (No. of OCys) was counted and normalized by bone area (mm^2^) in the alveolar bone between the distal root of the first molar and the mesial root of the second molar (*n* = 3 in each group). ^*^; *p* < .05 by *t*-test. (D and E) The number of canaliculi per OCy was counted. Primary canaliculi emanating from each lacuna and extending as a single, unbranched process were traced to determine canalicular length. The canalicular number and mean length were taken from at least 5 OCys per field in the alveolar bone between the distal root of the first molar and the mesial root of the second molar, with 3 fields per mouse (*n* = 3 in each group). ^*^; *p* < .05 by *t*-test.

To investigate age-related changes in the LCN in the alveolar bone, maxillae were harvested from 2-mo-old (young) and 16-mo-old (old) mice. The LCN was visualized using Ploton silver staining in both sexes. Prior to this experiment, the sample size was determined by power analysis using preliminary measurements obtained from young and old mice of both sexes (Figure S1). In both old male and female mice, reduced LCN coverage was observed in alveolar bone regions adjacent to the PDL (Figure 1A). The LCN area per bone area was quantified. In young mice, the LCN area per bone area was approximately 70%, whereas this ratio significantly decreased to about 50% in old mice (Figure 1B). The LCN area per bone area was similar between male and female mice (Figure 1B). Since age-related reductions in OCy numbers have been reported in the femur,^11,28^ the number of OCys per bone area was counted. A significant reduction in OCy density was observed in both old male and female mice (Figure 1C). In addition, the number and length of canaliculi per OCy were quantified, and both parameters were significantly lower in old mice (Figure 1D and E).

### More PDLCs expressed p16^INK4A^ and SASP factors in aged alveolar bones

**Figure 2:**
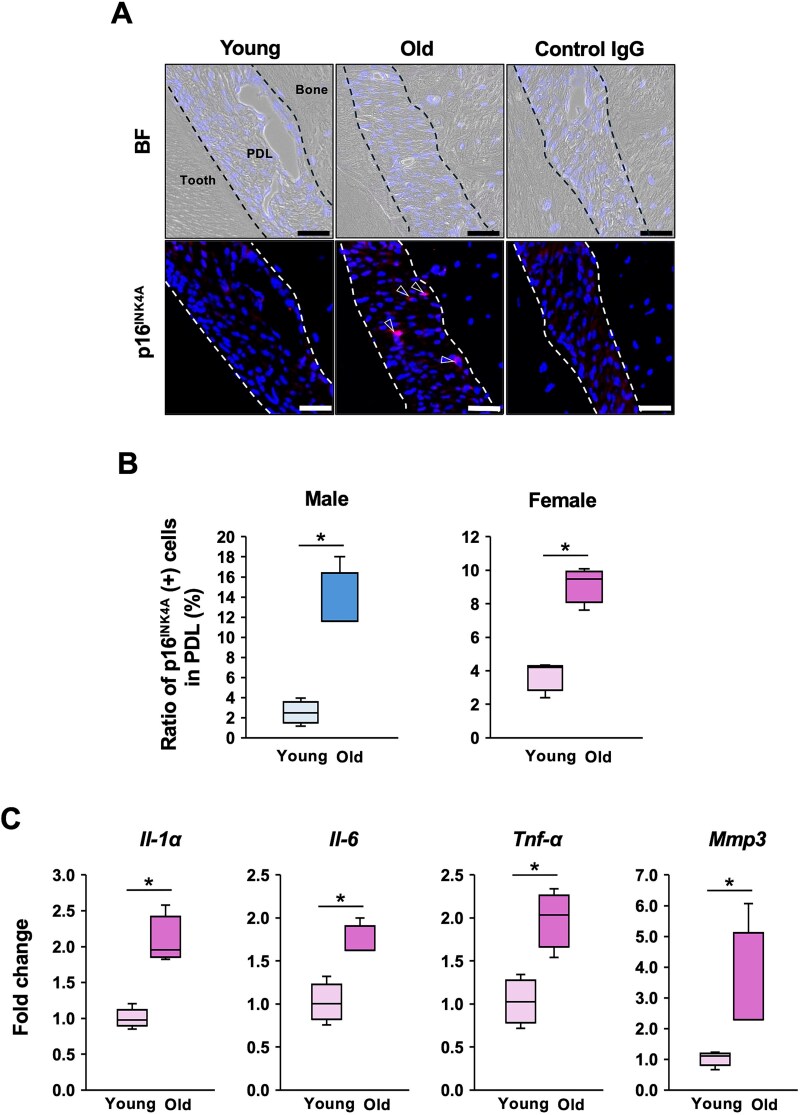
Senescence and SASP in the aged periodontal ligament. (A) Maxillary sections from 2-mo-old (young) and 16-mo-old (old) male and female mice were immunostained with anti-p16^INK4A^ antibody. Representative images of the PDL around the distal root of the first molar in female mice are shown. Arrowhead indicates a p16^INK4A^*-*positive cell. BF: bright field, PDL: periodontal ligament. Scale bars are 50 μm. (B) The ratio of p16^INK4A^*-*positive cells in the PDL was quantified around the distal root of the first molar (*n* = 3 in each group). The mean value from three randomly selected fields of view was obtained for each mouse. ^*^; *p* < .05 by *t*-test. (C) First and second maxillary molars were extracted, and the PDL attached around the teeth was corrected. Gene expressions related to SASP in female 1-mo-old (*n* = 4) and 14-mo-old mice (*n* = 3) were assessed by RT-qPCR. Fold changes from the average value of young mice were calculated by the ΔΔCT method. ^*^; *p* < .05 by *t*-test.

Recently, the localization of senescent cells in periodontal tissues, particularly in the PDL of aged mice, has been reported.^18^ Since p16^INK4A^ was considered a senescence marker associated with aging,^29^ maxillary sections from young and old mice were stained using a specific antibody against p16^INK4A^, which had been validated by the Mayo Clinic group.^30^ Representative images from female mice showed increased expression of p16^INK4A^ in the PDL adjacent to the alveolar bone in old mice (Figure 2A). The ratio of p16^INK4A^-positive cells per total cells in PDL was significantly higher in both male and female old mice compared to young mice (Figure 2B). To determine whether PDLCs exhibited a SASP, the first and second maxillary molars were extracted, and the PDL tissue was collected. The mRNA expression levels of SASP-related genes, including *Il-1α, Il-6, Tnf-α*, and *Mmp3*, were quantified by RT-qPCR. All of these SASP-associated mRNA expressions were significantly upregulated in PDLs from old mice compared to those from young mice (Figure 2C).

### SASP factors secreted by senescent PDLCs impaired dendrite formation by OCy-like cells in the 3D culture system

**Figure 3:**
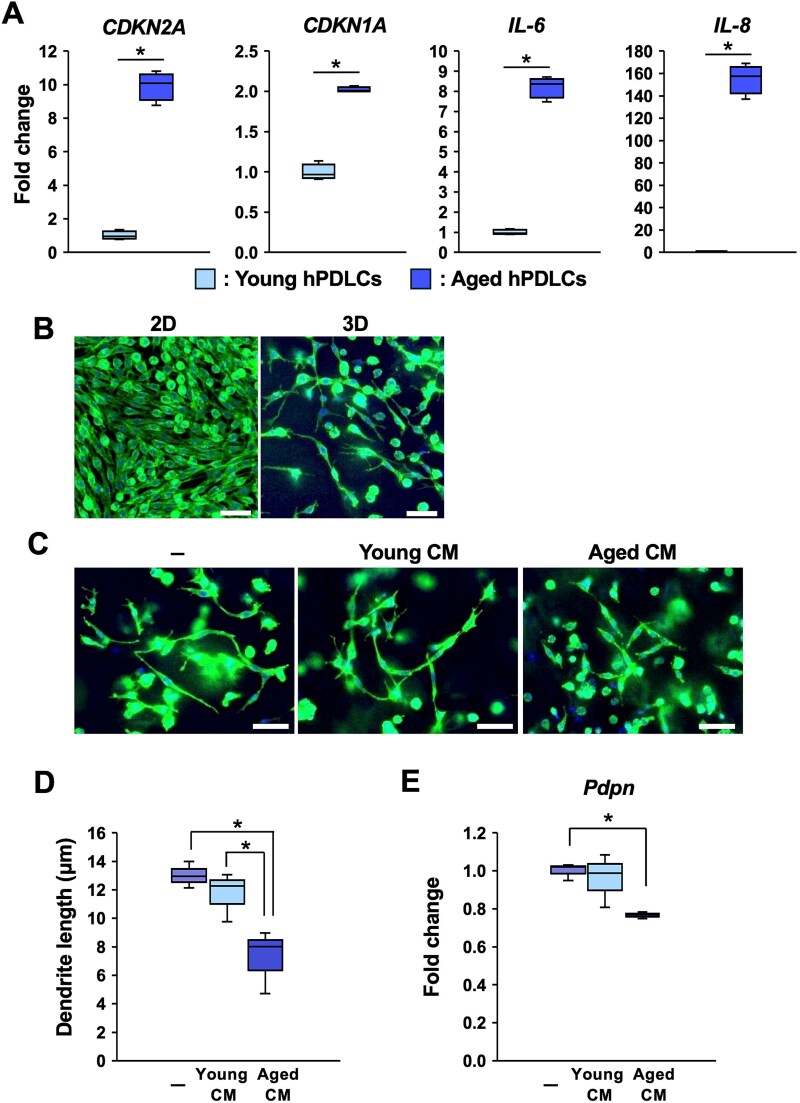
The effect of CM from aged PDLCs on dendrite formation in the 3D culture system. (A) Human PDLCs were serially passaged more than 20 times to induce cellular aging. Gene expressions related to SASP in young hPDLCs (passaged number 8) and aged hPDLCs (passaged number 23) were assessed by RT-qPCR. Fold changes from the average value of young hPDLCs were calculated by the ΔΔCT method. The data were obtained from three biological replicates. ^*^; *p* < .05 by *t*-test. (B) The murine osteocytic cell line MLO-Y4 was cultured on a 12-well plate (2D culture) or in a Bio-Block (3D culture) for 7 d. Representative images of phalloidin and Hoechst staining in 2D and 3D cultures are shown. Scale bars: 50 μm. (C) MLO-Y4 cells were cultured in the Bio-Block for 5 d to allow sufficient attachment, after which they were exposed to CM from young hPDLCs (passage numbers 5-9) or aged hPDLCs (passage numbers >20) at a concentration of 20% for 2 d. The cells were stained with iFluor 488-conjugated phalloidin and Hoechst. Representative images from three biological replicates are shown. Images were acquired at 200× magnification. Scale bars: 25 μm. (D) Dendrite length in the Bio-Block was quantified by tracing the longest process from each cell. At least 15 cells per field were analyzed, and the mean value from 3 randomly selected fields of view was obtained for each Bio-Block. The data represent three biological replicates. ^*^; *p* < .05 by Tukey’s test. (E) MLO-Y4 cells were cultured for 2 d in the presence or absence of young or aged CM. Gene expression of podoplanin (*Pdpn/E11*) was assessed by RT-qPCR. Fold changes relative to the average value of non-stimulated cells were calculated using the ΔΔCT method. Data were obtained from 3 biological replicates. ^*^; *p* < .05 by Tukey’s test.

Since inflammation impairs OCy maturation and dendrite formation,^22^ we hypothesized that SASP factors secreted by senescent PDLCs may contribute to the reduction of the LCN in aged alveolar bones. Human PDLCs were serially passaged more than 20 times to induce cellular aging, and senescent hPDLCs were generated as previously described.^18^ These aged hPDLCs expressed the senescence markers *CDKN2A* (*p16^INK4A^*) and *CDKN1A* (*p21^Cip1^*), as well as the SASP factors, *IL-6* and *IL-8*, confirming the senescent and SASP phenotypes of the cells (Figure 3A).

To determine whether the SASP factors from aged hPDLCs impaired OCy dendrite formation, we performed a 3D culture using Bio-Block produced by RONAWK.^27^ Since the block contained numerous micro-tunnels within the gel, soluble factors could reach the cells inside the block. This feature enabled us to examine the effects of CM after seeding the cells into the blocks. The OCy-like cell line MLO-Y4 was seeded onto 12-well plates or into the blocks and cultured for 7 d. The MLO-Y4 cells showed dendrite formation in the 3D culture conditions (Figure 3B). Conditioned medium from young hPDLCs had no effect on dendrite formation by MLO-Y4 cells, however, CM from aged hPDLCs suppressed dendrite formation in the 3D culture (Figure 3C). The dendrite length was significantly shorter in MLO-Y4 cells treated with CM from aged hPDLCs (Figure 3D). E11, encoded by the *podoplanin* (*Pdpn*) gene, was an essential factor for promoting dendrite formation.^31,32^ The mRNA expression of *Pdpn* was significantly downregulated by CM from aged hPDLCs (Figure 3E).

### Clearance of senescent cells preserved the LCN in the alveolar bone in aged mice

**Figure 4:**
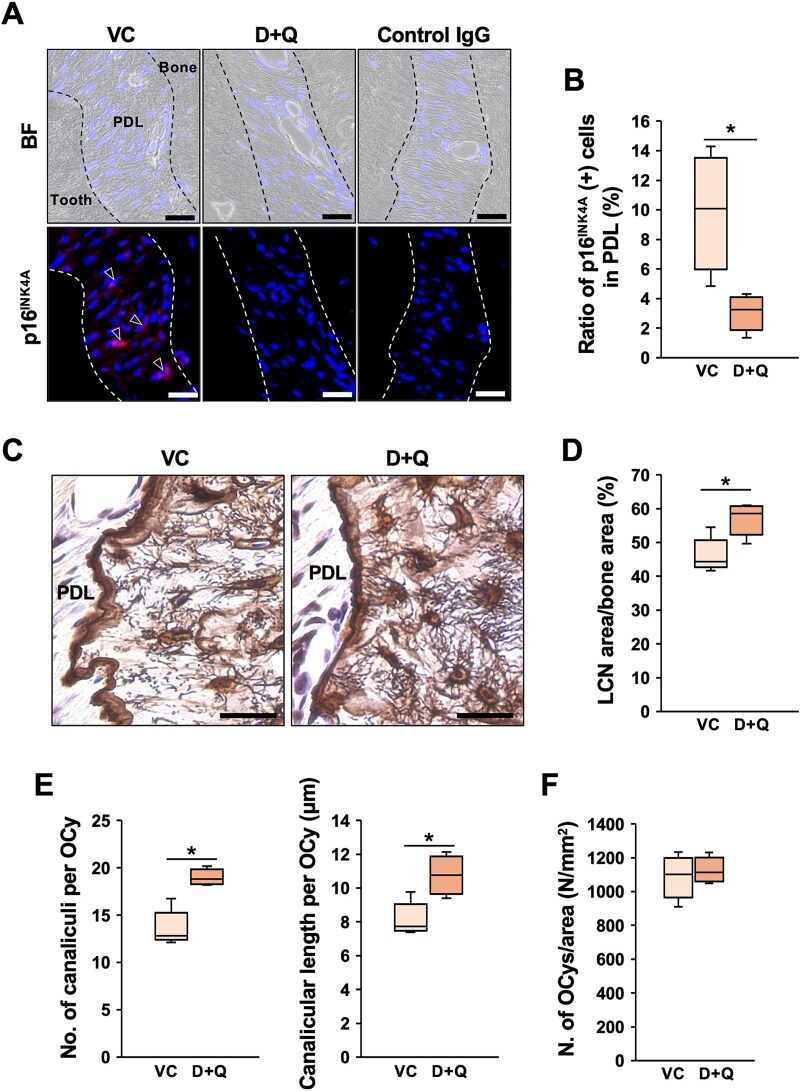
Alteration of the LCN in aged alveolar bone after senescent cell clearance. Twelve-month-old mice were randomly divided into a VC group and a D + Q-treated group. Mice were treated by oral gavage with D + Q or vehicle once per 2 weeks for 4 mo. (A) Maxillary sections from the VC group (*n* = 4) and the D + Q-treated group (*n* = 4) were immunostained with anti-p16^INK4A^ antibody. Representative images are shown. Arrowhead indicates a p16^INK4A^-positive cell. BF: bright field, PDL: periodontal ligament. Scale bars are 50 μm. (B) The ratio of p16^INK4A^*-*positive cells in the PDL around the distal root of the first molar was quantified in the VC group (*n* = 4) and the D + Q-treated group (*n* = 4). The mean value from three randomly selected fields of view was obtained for each mouse. ^*^; *p* < .05 by *t*-test. (C) Maxillary sections from the VC group (*n* = 4) and the D + Q-treated group (*n* = 4) were stained with Ploton silver staining. Representative images are shown. Scale bars are 50 μm. (D) The LCN area per bone area was quantified in the alveolar bone between the distal root of the first molar and the mesial root of the second molar in the VC group (*n* = 4) and the D + Q-treated group (*n* = 4). ^*^; *p* < .05 by *t*-test. (E) The number of canaliculi per OCy was counted. Primary canaliculi emanating from each lacuna and extending as a single, unbranched process were traced to determine canalicular length. The canalicular number and mean length were taken from at least 5 OCys per field in the alveolar bone between the distal root of the first molar and the mesial root of the second molar, with 3 fields per mouse in the VC group (*n* = 4) and the D + Q-treated group (*n* = 4). ^*^; *p* < .05 by *t*-test. (F) Number of OCys in the alveolar bone between the distal root of the first molar and the mesial root of the second molar was counted and normalized by bone area (mm^2^) in the VC group (*n* = 4) and the D + Q-treated group (*n* = 4). ^*^; *p* < .05 by *t*-test.

The results obtained so far suggested that SASP factors derived from senescent PDLCs may contribute to alterations in the LCN of aged alveolar bone. To further examine this concept, senescent cells were selectively cleared using a D + Q to suppress the SASP. D + Q has been widely used by many researchers for the systemic elimination of senescent cells.^23,33,34^ Since there was no significant reduction in LCN area per bone area at 12 mo of age (Figure S2), we hypothesized that the LCN alterations observed at 16 mo might begin developing from around 12 mo of age. Therefore, D + Q was orally administered to female mice for 4 mo, starting at 12 mo of age. The treatment period was determined based on the previous study.^23^ This treatment resulted in a marked reduction in the number of p16^INK4A^-expressing PDLCs compared to the VC group, indicating the effective clearance of senescent cells in the PDL (Figure 4A and B). In addition, the LCN in the maxillary alveolar bone appeared denser in D + Q-treated mice compared to VC (Figure 4C). Quantitative analysis revealed significant increases in the LCN area, as well as in the number and length of canaliculi per OCy, in the D + Q-treated group (Figure 4D and E). However, the total number of OCys per bone area was not significantly affected by the D + Q treatment (Figure 4F). In addition to the preservation of the LCN, osteoblast numbers and surfaces were increased, whereas those of osteoclasts were decreased in the alveolar bone of D + Q-treated mice (Figure S3).

### D + Q treatment altered E11 and sclerostin expression in aged alveolar bone

**Figure 5:**
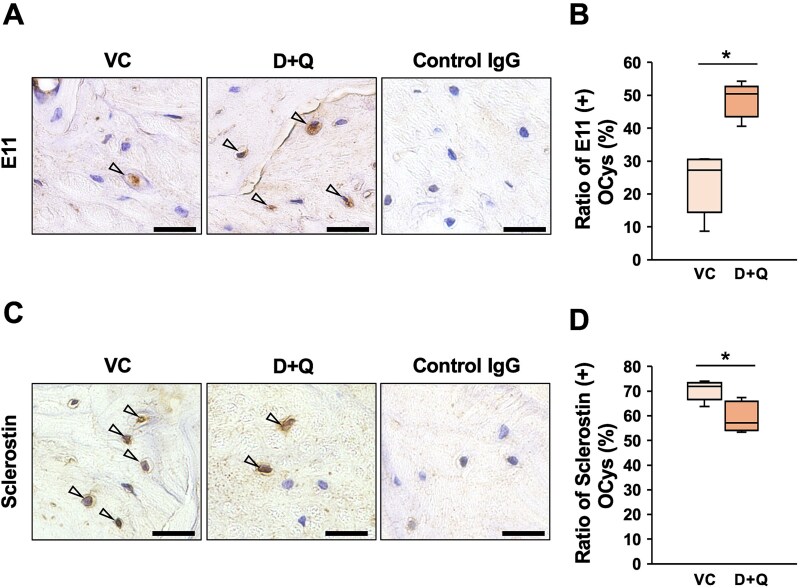
The expression of E11 and sclerostin in the alveolar bone following senescent cell clearance. Twelve-month-old mice were randomly divided into a VC group and a D + Q-treated group. Mice were treated by oral gavage with D + Q or vehicle once per two weeks for 4 mo. (A) Maxillary sections from the VC group (*n* = 4) and the D + Q-treated group (*n* = 5) were immunostained with anti-E11 antibody. Representative images are shown. Arrowhead indicates an E11-positive OCy. Scale bars are 25 μm. (B) The ratio of E11*-*positive OCys in the alveolar bone was quantified in the alveolar bone between the distal root of the first molar and the mesial root of the second molar in the VC group (*n* = 4) and the D + Q-treated group (*n* = 5). ^*^; *p* < .05 by *t*-test. (C) Maxillary sections from the VC group (*n* = 4) and the D + Q-treated group (*n* = 5) were immunostained with anti-sclerostin antibody. Representative images are shown. Arrowhead indicates a sclerostin-positive OCy. Scale bars are 25 μm. (D) The ratio of sclerostin*-*positive OCys in the alveolar bone was quantified in the alveolar bone between the distal root of the first molar and the mesial root of the second molar in the VC group (*n* = 4) and the D + Q-treated group (*n* = 5). ^*^; *p* < .05 by *t*-test.

Since CM from aged hPDLCs suppressed both dendrite formation and *Pdpn* mRNA expression in vitro (Figure 3), we hypothesized that E11 might be involved in the LCN preservation observed after D + Q treatment. To test this hypothesis, E11 expression was examined in both the VC and D + Q-treated groups. More OCys expressed E11 in the alveolar bone in the D + Q-treated group relative to the VC group (Figure 5A). The ratio of E11-positive OCys was quantified and found to be significantly increased by D + Q treatment (Figure 5B). To further examine the underlying mechanisms, the expression of sclerostin was assessed. Sclerostin was upregulated in inflammatory conditions^35^ as well as aging,^36^ and sclerostin was reported to downregulate E11 expression.^37^ There were significantly fewer sclerostin-expressing OCys in the alveolar bones of the D + Q-treated group (Figure 5C and D).

Perilacunar remodeling (PLR) is known to contribute to the maintenance of LCN integrity.^38^ Cathepsin K is one of the enzymes involved in PLR, and previous studies have shown that CTSK activity can be associated with structural changes in the LCN.^39,40^ The number of CTSK-positive OCys was significantly reduced in the alveolar bone of D + Q-treated mice (Figure S4).

## Discussion

Age-related alterations of the LCN and their relationship with bone metabolism have been reported in both animal and human studies of long bones. However, this topic has received little attention in the alveolar bone. To the best of our knowledge, this is the first report describing age-related changes in the LCN of the alveolar bone. The LCN of aged mice was significantly poorer than that of young mice, with a significantly reduced number and length of canaliculi. Similar to the aged long bones, the number and length of canaliculi, as well as the number of OCys, decreased in aged alveolar bones. These alterations were consistent in both sexes (Figure 1). Since a low density of the LCN in long bones has been reported to result in reduced bone metabolism, these age-related changes in the LCN may contribute to deterioration in the remodeling of the aged alveolar bone, such as increased susceptibility to infection,^6^ decreased bone volume,^7^ and diminished responsiveness to orthodontic forces.^8^ We used 16-mo-old mice as an aged model, although they are relatively younger than those used in previous studies.^11,23^ Previous studies on long bones have aimed to elucidate the mechanisms of age-related osteoporosis, which is commonly observed in people over 60 yr of age. In the dental field, the risk of periodontal disease increases around the age of 40^41^ and orthodontic tooth movement is limited after the growth period.^8^ These phenomena suggested that alterations in alveolar bone metabolism begin around mid-adulthood. Therefore, we selected 16-mo-old mice to mimic mid-adulthood and to examine whether age-related changes in the LCN contribute to these phenomena.

The PDL is a unique tissue located between the teeth and the alveolar bone. It contains various cell types, including PDLCs, immune cells, and stem/progenitor cells.^42^ The homeostasis of the PDL plays a critical role in maintaining alveolar bone remodeling, and PDL communicates closely with OCys.^43^ Recently, it was reported that an increased number of PDLCs exhibited senescence and produced inflammatory cytokines mediated by SASP.^18^ Since inflammation suppresses OCy dendrite formation,^22^ prolonged chronic inflammation in the periodontal microenvironment induced by SASP may contribute to the alteration of LCN in the alveolar bone. Consistent with a previous study,^18^ more PDLCs expressed p16^INK4A^ and SASP-related gene expressions in aged PDL (Figure 2). To examine whether the SASP factors produced by senescent PDLCs influenced the LCN, the effect of CM from senescent PDLCs was assessed on the dendrite formation by MLO-Y4 cells. Since it was difficult to observe the dendrite formation in the general 2D culture method,^44^ a 3D culture system using Bio-Block was employed. Conditioned medium from senescent PDLCs inhibited dendrite formation in the 3D culture system (Figure 3). E11 is an indispensable factor for stimulating dendrite formation. The critical role of E11 in maintaining bone homeostasis and responding to mechanical loading was demonstrated in E11/gp38 knockout mice, which exhibited a severely disrupted OCy dendritic network and a reduced mechanotransduction capacity.^32^ Conditioned medium from aged PDLCs downregulated *Pdpn/E11* expression, suggesting that SASP factors from senescent PDLCs suppressed dendrite formation by downregulating *Pdpn/E11* (Figure 3). However, it remains unclear which specific factors within the CM are essential for the suppression of dendrite formation in the 3D culture.

To investigate whether reducing SASP factors could attenuate age-related alterations in the LCN of aged alveolar bone, the senolytic cocktail consisting of D + Q was administered. Treatment with D + Q decreased p16^INK4A^-expressing PDLCs in aged PDL, suggesting the clearance of senescent PDLCs and a reduction in SASP factors. Lacunar-canalicular network integrity in aged alveolar bone was significantly improved in D + Q-treated mice compared to the VC group (Figure 4). Since 12-mo-old mice were treated with D + Q for 4 mo, these findings suggest that age-related LCN deterioration was attenuated by the clearance of senescent cells from the periodontal microenvironment. These results indicated that prolonged exposure to SASP factors from senescent PDLCs may contribute to LCN degradation in aged alveolar bone. To explore the underlying mechanisms, the expression levels of E11 and sclerostin were examined by immunohistochemistry in vehicle- and D + Q-treated mice. After 4 mo of D + Q administration, E11 expression was upregulated, while sclerostin expression was downregulated (Figure 5). This increase in E11 expression was consistent with the in vitro data (Figure 3), supporting the hypothesis that E11 was suppressed by SASP factors derived from senescent PDLCs. Sclerostin, a negative regulator of bone formation produced by OCys, is known to be upregulated by inflammation and aging.^35,36^ In DMP1-GsαKO mice, which exhibited an osteopenic phenotype, sclerostin antibody treatment markedly increased the E11/gp38 positive OCys near the endosteal bone and endosteal osteoblasts.^37^ This report suggested that sclerostin caused the downregulation of E11 expression. Sclerostin expression was downregulated by D + Q treatment, and E11 expression was upregulated in the same mice (Figure 5), suggesting the existence of a SASP-sclerostin upregulation-E11 downregulation axis in aged alveolar bone, in addition to the direct suppression of E11 expression by SASP factors. Furthermore, sclerostin inhibits new bone formation and osteoblast-to-osteocyte differentiation; thus, the downregulation of sclerostin expression by D + Q treatment may also be beneficial for maintaining alveolar bone homeostasis. In fact, D + Q treatment increased the number and surface of osteoblasts while reducing those of osteoclasts (Figure S3). Because defects in osteoblast-lineage cells lead to reduced bone formation and abnormal OCy networks,^45^ the increased number of osteoblasts observed in D + Q-treated aged alveolar bone may also have contributed to improving LCN parameters by supplying new, E11-positive OCys. Cathepsin K expression in OCys has been reported to be associated with alterations in the LCN through changes in PLR activity.^39,40^ D + Q treatment also decreased the number of CTSK-expressing OCys (Figure S4), suggesting that modulation of PLR-related responses may have contributed to the preservation of LCN integrity in D + Q-treated mice.

However, a study by Farr et al. reported that OCy senescence and its associated SASP factors contributed to age-related bone loss in the femur.^46^ This suggests that SASP factors derived from senescent OCys, in addition to those from senescent PDLCs, may be involved in age-related changes in the LCN. Since D + Q treatment induces the systemic clearance of senescent cells, it is possible that SASP factors from other senescent cell types may also influence LCN alterations in aged alveolar bone. Although our findings from the 3D culture system (Figure 3) support the hypothesis that SASP factors from senescent PDLCs impair OCy dendrite formation, this remains a limitation of the current study. Future studies employing transgenic animal models that allow for the specific and localized clearance of senescent PDLCs using approaches similar to those demonstrated in a previous research^47^ would provide deeper insight. Another limitation of this study is the relatively small number of animals included. Sample sizes were determined by power analysis to ensure the minimum necessary use of animals in line with animal welfare principles.^48^ The sample sizes were sufficient to achieve statistical validity; however, the small number of animals may still limit the interpretation of some secondary analyses. Further studies with larger sample sizes and more advanced three-dimensional characterization of the LCN in the alveolar bone would be essential to substantiate and extend the conclusions of this research.

Taken together, these findings suggest that SASP factors secreted by senescent PDLCs contribute to the age-related deterioration of the LCN in alveolar bone, potentially impairing OCy function and disrupting bone remodeling homeostasis. Our in vitro and in vivo data indicate that the inflammatory microenvironment associated with aging, shaped by senescent PDLCs, negatively impacts OCy dendrite formation, partly through the downregulation of E11 expression. These results emphasize the importance of the PDL as a regulatory tissue influencing alveolar bone quality during aging and highlight PDLC senescence as a potential therapeutic target. Targeting senescent PDLCs may be a promising strategy to preserve OCy integrity and maintain alveolar bone homeostasis in an aging population.

## Supplementary Material

ziag014_Supplemental_File

## Data Availability

The data underlying this article are available in the article and in its online supplementary material.
