# Supplementary material for: Inflammatory cytokines secreted from senescent periodontal ligament cells influence the osteocyte network in alveolar bone
Source: JBMR Plus. 2026 Jan 29;10(3):ziag014. doi: 10.1093/jbmrpl/ziag014 (PMC12908679; doi:10.1093/jbmrpl/ziag014)
Supplement: ziag014_Supplemental_File [file ziag014_supplemental_file.pdf]

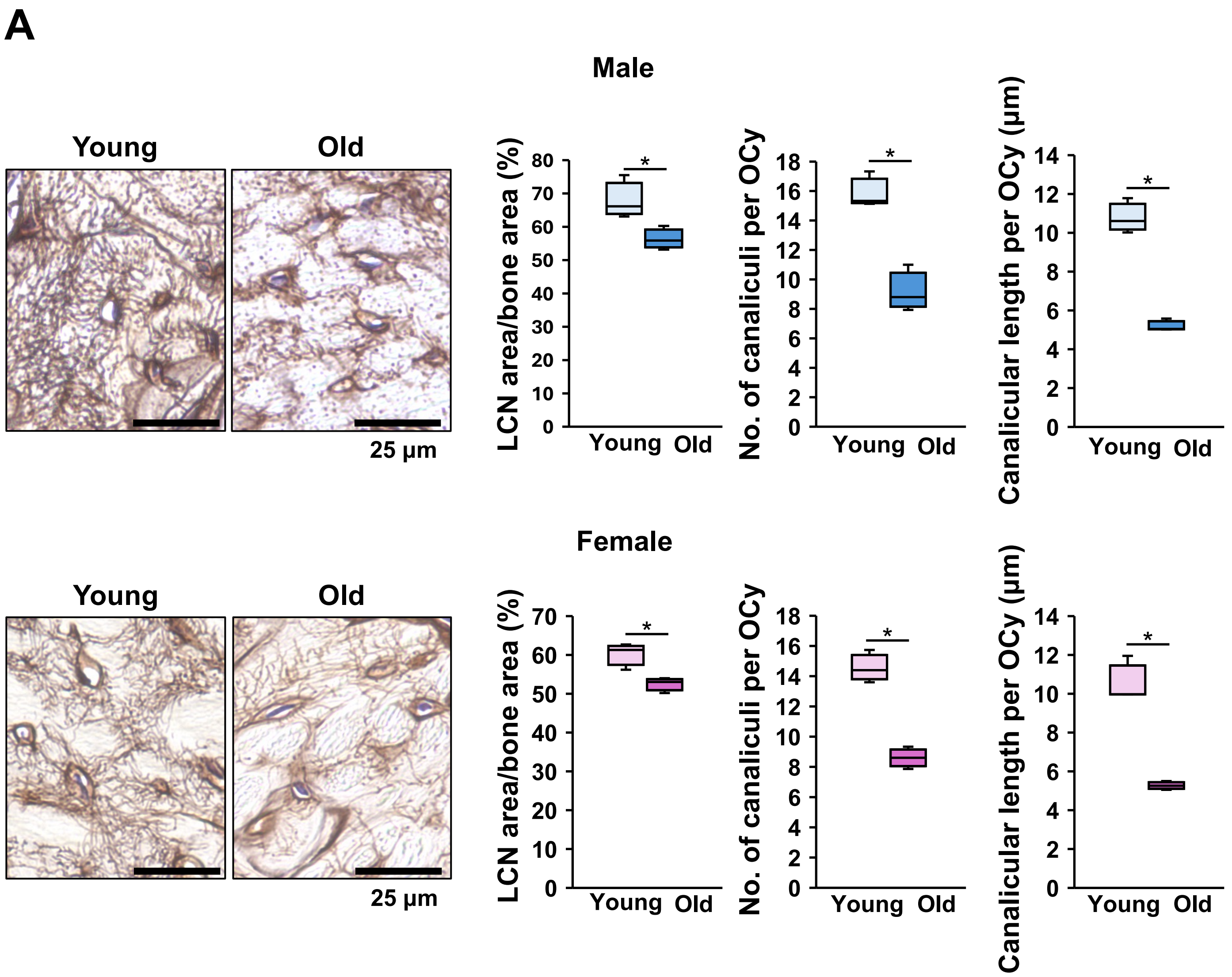

B

		LCN area/bone area (average, %)	SD	n	Effect size (Cohen's d)	α	Power (1-β)	Sample size (n)
Male	Young	68.2	6.5	3	2.8	0.05	0.8	3.0
	Old	56.4	3.6	3				
Female	Young	60.0	3.4	3	3.4	0.05	0.8	2.4
	Old	52.4	2.0	3				

Supplementary Figure 1. Preliminary measurements used to determine sample sizes in the main study. (A) Maxillary sections derived from 2-month-old (young) and 18-month-old (old) male mice or 2-month-old (young) and 19-month-old (old) female mice were stained with Ploton silver staining. The ratio of LCN area per bone area (%), the number of canaliculi per OCy, and the canalicular length per OCy (µm) were quantified in the alveolar bone between the distal root of the first molar and the mesial root of the second molar (n = 3 in each group). *; $P < 0.05$ by *t*-test. **(B)** The sample size for the main study was determined by power analysis using the results of LCN area per bone area.

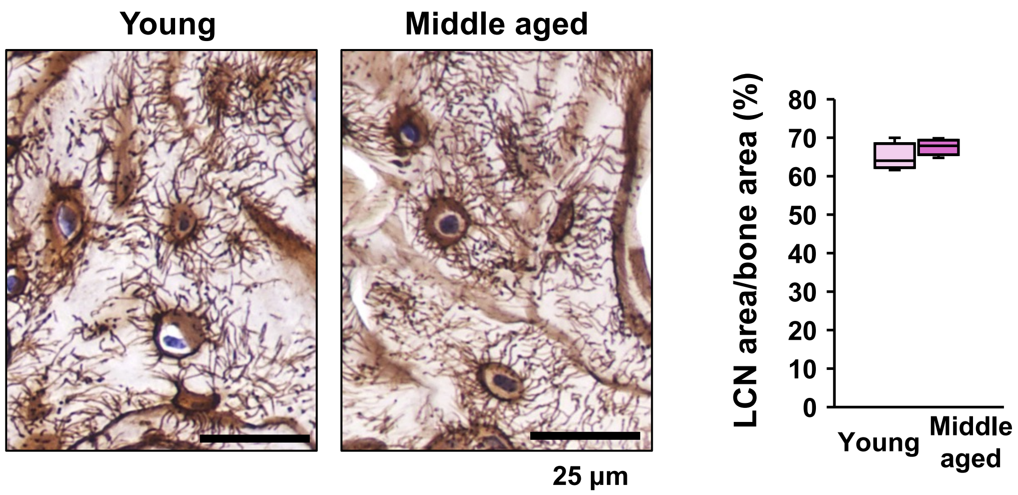

Supplementary Figure 2. LCN in the alveolar bone from young and middle-aged mice. Maxillary sections derived from 2-month-old (young) and 12-month-old (middle aged) female mice were stained with Ploton silver staining. The ratio of LCN area per bone area was quantified in the alveolar bone between the distal root of the first molar and the mesial root of the second molar (n = 3 in each group). *; $P < 0.05$ by *t*-test.

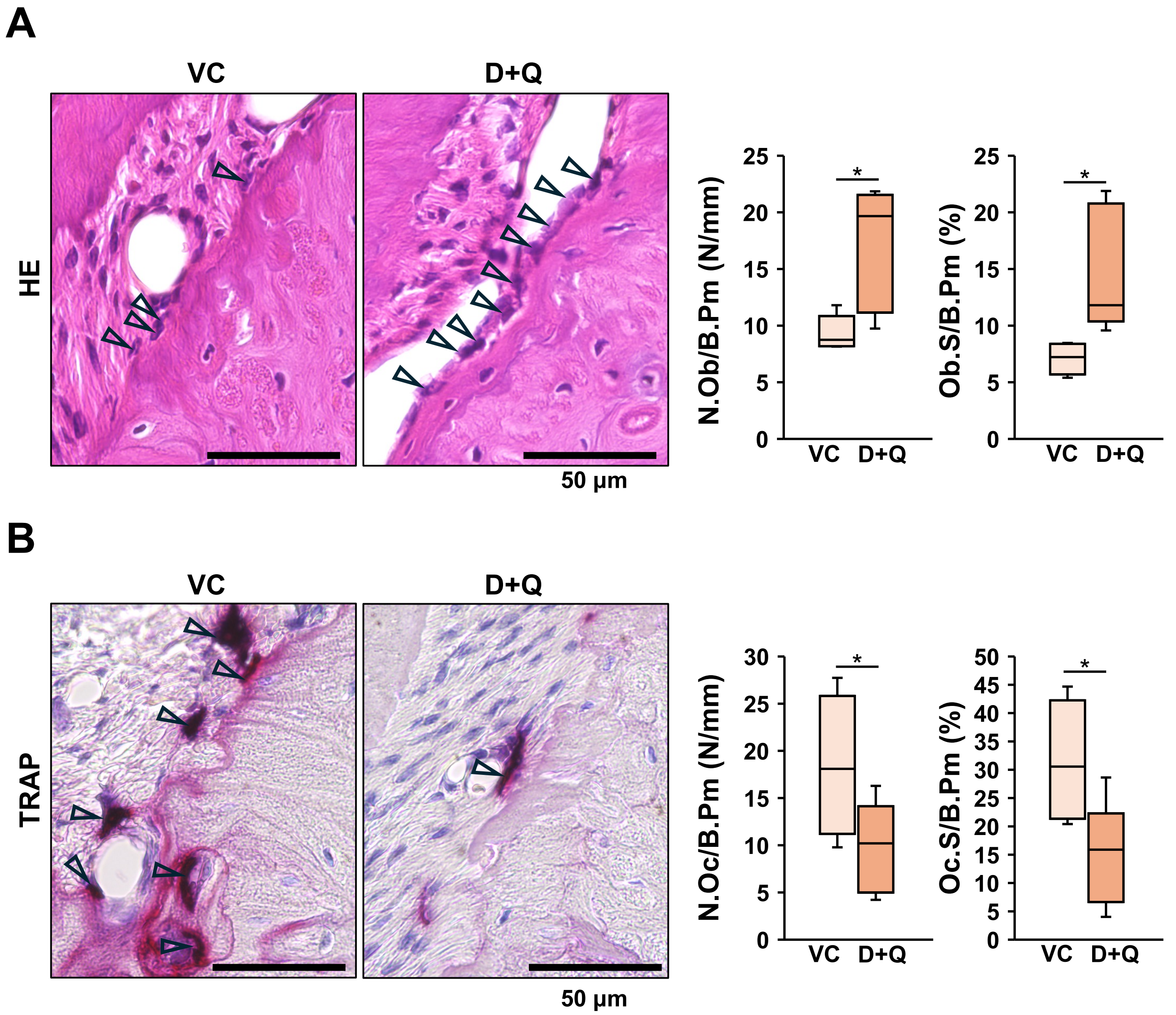

Supplementary Figure 3. The effects of D + Q on osteoblasts and osteoclasts in the alveolar bone. (A) Maxillary sections derived from 16-month-old vehicle-treated mice (VC, n = 4) and D + Q-treated mice (D + Q, n = 5) were stained with H&E. Number of osteoblasts per bone perimeter (N.Ob/B.Pm) and osteoblast surface per bone perimeter (Ob.S/B.Pm) were quantified on the alveolar bone between the distal root of the first molar and the mesial root of the second molar. Arrowhead indicates osteoblast. *; $P < 0.05$ by *t*-test. **(B)** Maxillary sections derived from VC group (n = 4) and D + Q group (n = 5) were stained with TRAP. Number of osteoclasts per bone perimeter (N.Oc/B.Pm) and osteoclast surface per bone perimeter (Oc.S/B.Pm) were quantified on the alveolar bone between the distal root of the first molar and the mesial root of the second molar. Arrowhead indicates osteoclast. *; $P < 0.05$ by *t*-test.

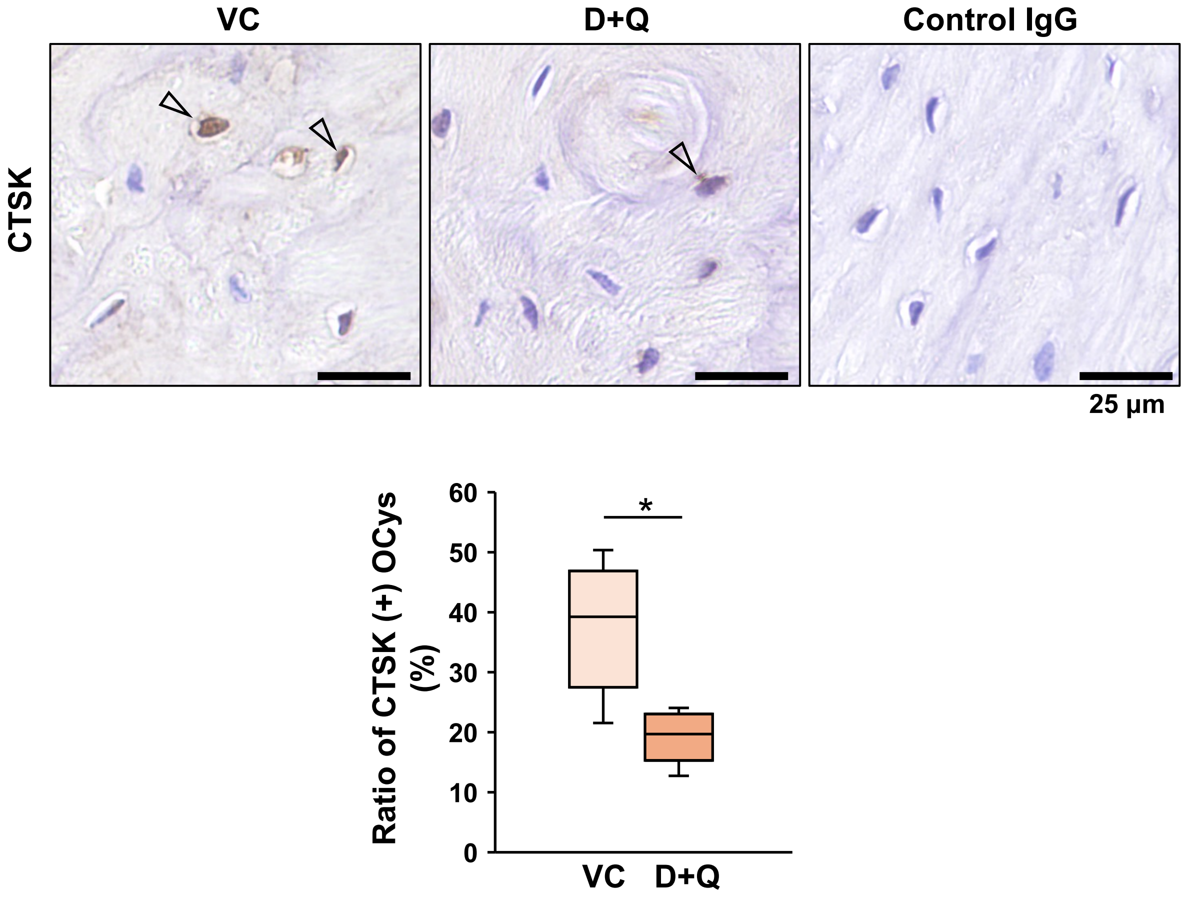

Supplementary Figure 4. The expression of CTSK on OCys in the alveolar bone after D + Q treatment. Maxillary sections from the VC group (n = 4) and the D + Q-treated group (n = 5) were immunostained for CTSK. Representative images are shown. Arrowhead indicates CTSK-positive OCys. The ratio of CTSK-positive OCys per total OCys was quantified in the alveolar bone between the distal root of the first molar and the mesial root of the second molar. *; $P < 0.05$ by *t*-test.

Supplemental table 1. Primer list

	**forward**	**reverse**
Mouse primers		
Il-1α	AAGAGACCATCCAACCCAGATC	CCTGACGAGCTTCATCAGTTTG
Il-6	ACCACGGCCTTCCCTACTTC	TTGGGAGTGGTATCCTCTGTGA
Tnf-α	ATGAGCACAGAAAGCATG	AGTAGACAGAAGAGCGTGGT
Mmp3	ACTCTACCACTCAGCCAAGG	TCCAGAGAGTTAGACTTGGTGG
Pdpn	ACCCCAATAGAGATGGCTTGC	AGCTCTTTAGGGCGAGAACC
Hprt	CGTGATTAGCGATGATGAACCA	TCCAAATCCTCGGCATAATGA
Human primers		
CDKN2A	CCTTTGGTTATCGCAAGCTG	CCCTGTAGGACCTTCGGTGA
CDKN1A	TCAAATCGTCCAGCGACCTTC	CATGCCCTGTCCATAGCCTCTAC
IL-6	GCCAGAGCTGTGCAGATGAG	TCAGCAGGCTGGCATTTG
IL-8	ATGACTTCCAAGCTGGCCGTGGCT	TCTCAGCCCTCTTCAAAAACTTCTC
HPRT	GGCAGTATAATCCAAAGATGGTCAA	GTCAAGGGCATATCCTACAACAAAC
